# Glaucoma and cardiovascular disease: a bidirectional two-sample Mendelian randomization analysis

**DOI:** 10.3389/ebm.2025.10610

**Published:** 2025-10-15

**Authors:** Dongdong Jin, Jie Sun, Wei Zhang, Mingxuan Zhang, Chengfang Li

**Affiliations:** ^1^ Department of Anesthesiology, Qingdao Traditional Chinese Medicine Hospital (Qingdao Hiser Hospital), Qingdao, Shandong, China; ^2^ Operating Room Nursing Department, Qingdao Traditional Chinese Medicine Hospital (Qingdao Hiser Hospital), Qingdao, Shandong, China; ^3^ Disinfection Supply Center, Qingdao Traditional Chinese Medicine Hospital (Qingdao Hiser Hospital), Qingdao, Shandong, China; ^4^ Department of Anesthesia Surgery, Qingdao Traditional Chinese Medicine Hospital (Qingdao Hiser Hospital), Qingdao, Shandong, China; ^5^ Department of Ophthalmology, Qingdao Traditional Chinese Medicine Hospital (Qingdao Hiser Hospital), Qingdao, Shandong, China

**Keywords:** glaucoma, cardiovascular disease, causal relationship, Mendelian randomization analysis, genetic correlation

## Abstract

Many studies reported that glaucoma is associated with cardiovascular disease (CVD). This study aims to investigate the potential causal relationship between glaucoma and CVD using a bidirectional two-sample Mendelian randomization (MR) analysis. The genome-wide association studies (GWAS) of glaucoma and CVD were downloaded from the IEU OpenGWAS project. The CVD included unstable angina pectoris (UAP), coronary artery disease (CAD), high blood pressure (HBP), myocardial infarct (MI), heart failure (HF), ischemic stroke (IS), atrial fibrillation (AF), and pulmonary embolism (PE). The inverse variance weighting (IVW) analysis was the primary method in MR analysis. Meanwhile, sensitivity analysis and statistical power tests were performed. The random effects IVW method showed a causal relationship between glaucoma and a decreased risk of MI (Odds ratio (OR): 0.94, 95% confidence interval (CI): 0.89–0.99; P = 0.012). In the reverse MR analysis, genetic susceptibility of UAP (OR: 1.12, 95% CI: 1.02–1.23; P = 0.022), CAD (OR: 1.1, 95% CI: 1–1.21; P = 0.041), and HBP (OR: 1.83, 95% CI: 1.25–2.67; P = 0.002) was significantly linked to an increased risk of glaucoma. MR-Egger (P = 0.005) and IVW (P = 0.005) methods found that HBP presented different degrees of heterogeneity. The random effects IVW method also demonstrated that HBP is the risk factor for glaucoma (P = 0.0017). Although reverse MR initially suggested a potential association between CAD and glaucoma, MVMR showed no causal relationship after adjusting for obesity and BMI. The MR analysis found that glaucoma serves as a protective factor for MI, while UAP and HBP were risk factors for glaucoma in the European population, which may contribute to preventing and managing glaucoma and CVD.

## Impact statement

Many studies reported that glaucoma is associated with CVD. However, the causal relationship between glaucoma and CVD is not clear. This discovery bridges the gap between basic research and clinical care, providing genetic-based evidence. This evidence could potentially guide the development of prevention strategies in the clinical practice of glaucoma and CVD, such as early screening for at-risk patients with specific cardiovascular conditions for glaucoma and *vice versa*, and tailoring treatment plans considering the identified causal relationships. The MR analysis found that glaucoma serves as a protective factor for MI, while UAP and HBP were risk factors for glaucoma in the European population, which may contribute to preventing and managing glaucoma and CVD.

## Introduction

Glaucoma is a chronic, progressive optic neuropathy, characterized by optic nerve damage and visual field loss, leading to irreversible blindness [1]. In 2020, glaucoma (3.6 million cases) is the second leading cause of blindness in people aged 50 and older worldwide, after cataracts (15.2 million cases) [2]. The global prevalence of glaucoma is 3.54%, and patients with glaucoma are expected to reach 111 million by 2040 [3]. Based on the anatomical state of the anterior chamber angle, glaucoma can be categorized into open-angle and closed-angle types [4]. Each type can be further divided into primary and secondary glaucoma [4]. An increase in intraocular pressure (IOP) is closely associated with the development of glaucoma, and lowering IOP is the only feasible way to treat glaucoma [1, 5]. However, the IOP of nearly 50% of glaucoma patients falls within the normal range [1]. Many studies have reported that there are associations between gut microbiota, body mass index, and waist circumference with glaucoma [6, 7]. Noteworthily, some cardiovascular diseases (CVD) have also been associated with glaucoma [8, 9].

CVD are various diseases that affect the heart and blood vessels, such as unstable angina pectoris (UAP), coronary artery disease (CAD), high blood pressure (HBP), myocardial infarction (MI), heart failure (HF), ischemic stroke (IS), atrial fibrillation (AF), and pulmonary embolism (PE) [10, 11]. CVD poses a heavy medical burden in China [12]. Several large population studies have reported CVD as a risk factor for open-angle glaucoma (OAG) [13, 14]. Bennion et al. demonstrated that heart disease was the leading cause of death in glaucoma-related mortality in the United States, followed by malignant neoplasms and cerebrovascular disease [15]. Similarly, patients with OAG are about 1.5 times more likely to develop CVD than non-glaucoma individuals [16]. However, the causal relationship between glaucoma and CVD is not clear.

Mendelian randomization (MR) analysis is applied to explore the potential causal relationship between exposure and outcome through single‐nucleotide polymorphisms (SNPs) as the instrumental variable (IV) [17]. Since the selected SNPs were randomly assigned through meiosis, MR results are not influenced by confounders between exposure and outcome and reverse causation [18]. Meng et al. reported that ankylosing spondylitis was the risk factor for both primary OAG and primary angle-closure glaucoma [19]. In addition, getting up easily in the morning and sleep duration were demonstrated as risk factors for POAG through MR analysis [20]. Therefore, the potential causal effects between glaucoma and CVD, including MI, HBP, HF, PE, CAD, UAP, IS, and AF, are explored using bidirectional two-sample MR analysis in this study.

## Materials and methods

### Data source and study design

Summary statistics data for glaucoma- and CVD-associated SNPs were collected from the IEU OpenGWAS project^1^. The GWAS of glaucoma^2^ included 8,591 cases and 210,201 controls. For the CVD datasets, summary data for UAP^3^ included 9,481 cases and 446,987 controls [21], summary data for CAD^4^ comprised 42,096 cases and 361 controls [22], summary data for HBP^5^ included 124,227 cases and 337,653 controls, summary data for MI^6^ comprised 20,917 cases and 440,906 controls [21], summary data for HF^7^ included 47,309 cases and 930,014 controls [23], summary data for IS^8^ comprised 11,929 cases and 472,192 controls [21], and summary data for AF^9^ contained 60,620 cases and 970,216 controls [24]. The summary data for PE^10^ included 407,746 samples [25]. The detailed information of all the GWAS is demonstrated in Table 1. All statistical data were gathered from the descendants of Europeans, reducing the potential bias due to racial differences. Notably, no overlap was observed, given that samples of glaucoma and CVD originated from distinct study groups.

**TABLE 1:** Detailed information on the GWAS datasets included in this study.

Exposure or outcomes	GWAS ID	Sample size (case/controls)	SNPs	Year
Glaucoma	finn-b-H7_GLAUCOMA	8,591/210,201	16,380,466	2021
Unstable angina pectoris	ebi-a-GCST90018932	9,481/446,987	24,179,929	2021
Coronary artery disease	ebi-a-GCST003116	42,096/361	8,597,751	2015
High blood pressure	ukb-b-14177	124,227/337,653	9,851,867	2018
Myocardial infarct	ebi-a-GCST90018877	20,917/440,906	24,172,914	2021
Heart failure	ebi-a-GCST009541	47,309/93,0014	7,773,021	2020
Ischemic stroke	ebi-a-GCST90018864	11,929/472,192	24,174,314	2021
Atrial fibrillation	ebi-a-GCST006414	60,620/970,216	11,039,197	2018
Pulmonary embolism	ebi-a-GCST90013887	407,746	33,519,037	2021

SNPs: Single Nucleotide Polymorphisms.

**FIGURE 1:**
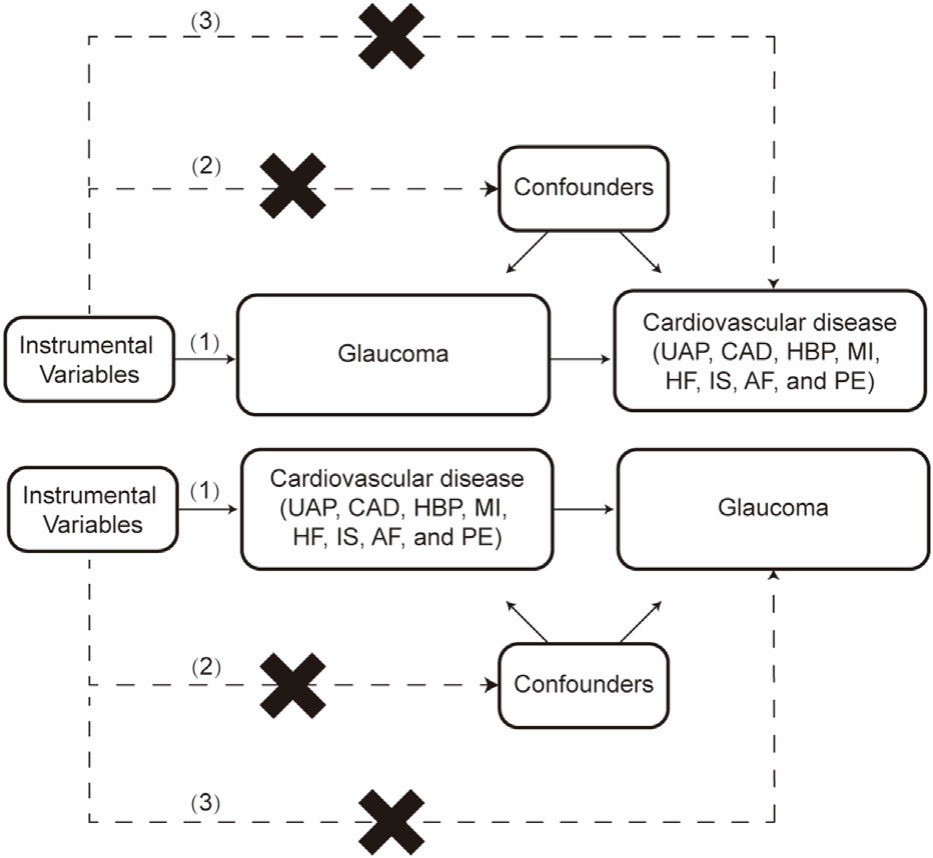
The flow chart of this study. The MR method is based on 3 hypotheses: (1) Instrumental variables (IVs) must be closely related to the exposure; (2) IVs independent from confounders; (3) IVs only affect outcomes through exposure, not through other pathways. UAP: Unstable angina pectoris, CAD: Coronary artery disease, HBP: High blood pressure, MI: Myocardial infarction, HF: Heart failure, IS: Ischemic stroke, AF: Atrial fibrillation, PE: Pulmonary embolism.

Two-sample MR analysis was utilized to explore the potential causal association between glaucoma and CVD risks. An overview of the study design is shown in Figure 1. SNPs served as IVs must satisfy the following conditions: (1) IVs must be closely related to the exposure; (2) IVs independent from confounders; (3) IVs only affect outcomes through exposure, not through other pathways.

### Selection of IVs

First, the SNPs with *p*-value < 5 × 10^−8^ were selected as the IVs. Then, the parameters (r^2^ < 0.001 and clump window = 10,000 kb) were utilized to remove the linkage disequilibrium bias. Next, SNPs with F-statistic >10, strongly correlated with exposure, were obtained [26].

### Statistical analysis

This study used three methods (fixed-effects inverse variance weighted (IVW), weighted median, and MR-Egger) to perform MR analysis. The fixed-effects IVW method is the primary analysis to assess the causal associations between glaucoma and CVD according to heterogeneity [27]. Weighted median and MR-Egger methods were applied as supplementary analyses. The weighted median method can provide reliable causal estimates when at least half of the IVs are valid [28]. MR-Egger regression can discern horizontal pleiotropy and offer causal estimates after correction for horizontal pleiotropy [28].

For sensitivity analysis, firstly, MR-Egger regression and IVW methods were used to detect heterogeneity [28]. Cochrane’s Q-derived *p*-value < 0.05 indicated that heterogeneity existed between IVs, and then random effects IVW was carried out to calculate causal estimates [29]. Second, for the horizontal pleiotropy analysis, the MR-Egger intercept was performed. The *p*-value > 0.05 was considered a weak possibility of genetic pleiotropy, and its impact may be discarded [30]. The MR-PRESSO method was utilized to remove outliers and then calculate causal estimates [31]. Finally, leave-one-out was performed to evaluate whether the results were caused by a single SNP [32]. The “TwoSampleMR” and “MR-PRESSO” packages in R (v 4.2.3) were utilized to perform statistical analysis.

### Summary data-based mendelian randomization (SMR) analysis

Using the summary statistics of eQTL and GWAS, the association between gene expression and HBP and glaucoma was tested by SMR analysis. Gene expression was used as the exposure factor, and HBP and glaucoma were the outcome factors. The SNPs of cis-eQTL were used as IVs. The analysis was performed using Rstudio and SMR software, with default settings applied during the analysis. Genes with significant loci were selected using P_SMR less than 0.05, and the heterogeneity in dependent instruments (HEIDI) test was used to evaluate the heterogeneity of the results (p_HEIDI > 0.05). Subsequently, GO and KEGG enrichment analysis (P < 0.05) was performed and a Protein-Protein Interaction (PPI) network was constructed to explore the possible molecular mechanisms involved in potential pathogenic genes.

## Results

### Information of IVs

A total of 11 SNPs associated with glaucoma were obtained. For SNPs linked to CVD, there were 14 for UAP, 31 for CAD, 187 for HBP, 67 for MI, 9 for HF, 13 for IS, 7 for PE, and 97 for AF. All SNPs were satisfied the following criteria: p < 5 × 10^−8^, r^2^ < 0.001, and clump window = 10,000 kb. Simultaneously, palindromic SNPs were removed. The range of SNPs associated with glaucoma based on F-statistic was 30.91–135.09, while the range of SNPs linked to CVD was 29.50–2039.47.

### Causal effects of glaucoma on CVD

**FIGURE 2:**
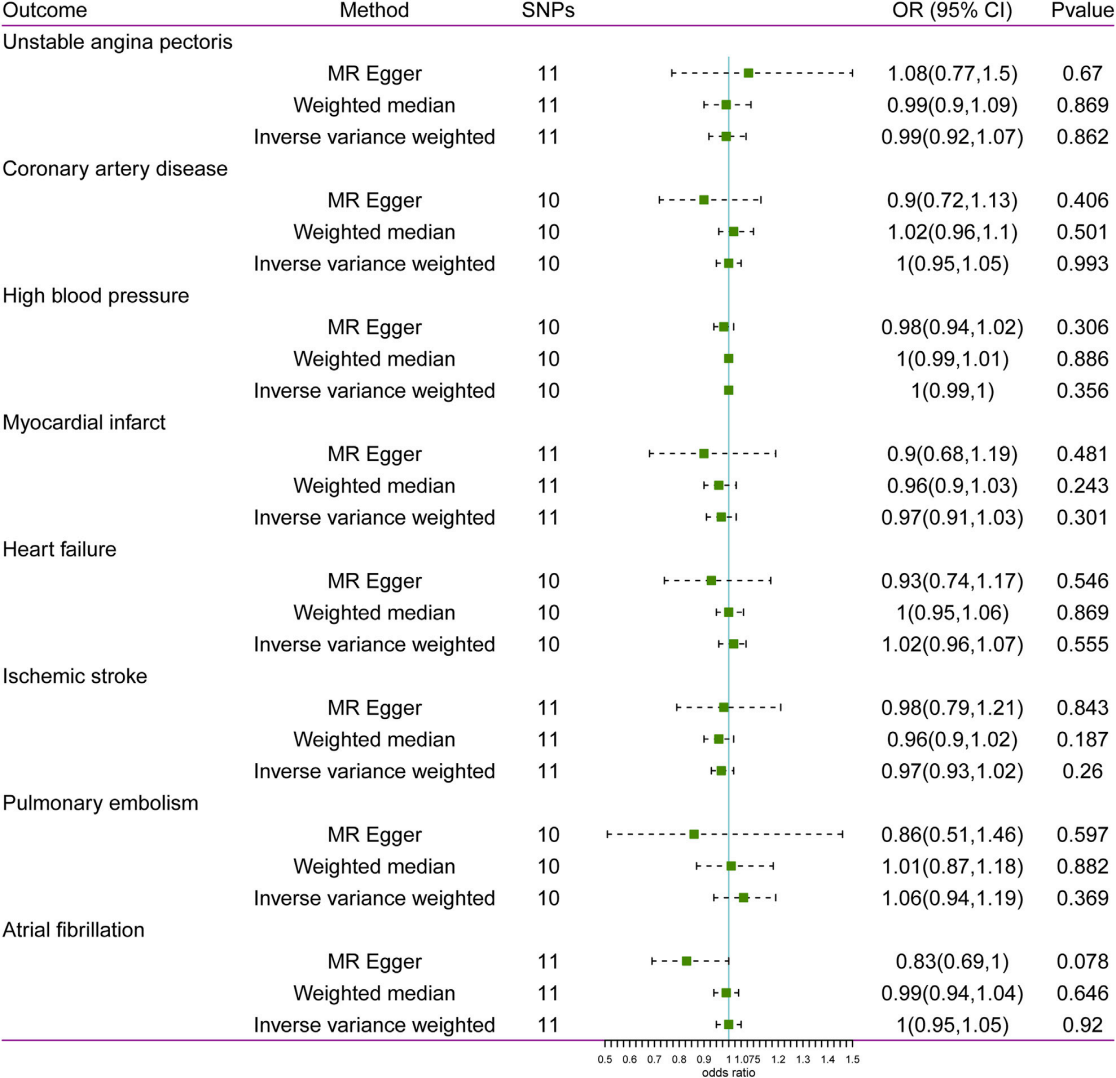
Associations between glaucoma and risk of cardiovascular disease using Mendelian randomization analysis. SNPs: Single-nucleotide polymorphisms, OR: Odds ratio, CI: Confidence interval.

**FIGURE 3:**
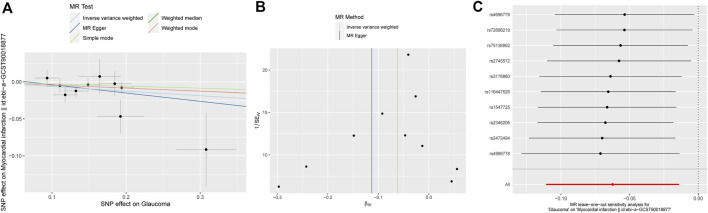
Associations between glaucoma and risk of MI using Mendelian randomization analysis. **(A)** Scatter plot for the associations between glaucoma and MI. **(B)** Funnel plots for the relationship between glaucoma and MI. **(C)** Leave-one-out for the relationship between glaucoma and MI. MI: Myocardial infarction; SNP: Single nucleotide polymorphism.

The results of MR analysis do not support a causal relationship between glaucoma and CVD risks (Figure 2). The *p*-values of Cochran’s Q test were less than 0.05 for both MR-Egger and IVW methods in HBP and MI, demonstrating that the IVs of HBP and MI presented different degrees of heterogeneity (Table 2). Additionally, IVW analysis showed that IVs in AF (P = 0.025) also have heterogeneity (Table 2). Meanwhile, the MR-Egger regression intercept test found that IVs among CVD were not present with horizontal pleiotropy (Table 2). Then, random effects IVM analysis was carried out, and we found that no causal associations existed between glaucoma and HBP (P = 0.356), MI (P = 0.301), and AF (P = 0.92). MR-PRESSO analysis showed that HBP (P < 0.001), MI (P = 0.049), and AF (P = 0.021) existed horizontal pleiotropy. After removing outliers, causal relationships between glaucoma and HBP, as well as AF, were not present. However, there was a causal relationship between glaucoma and MI (P = 0.028). Subsequently, MR analysis was performed again after removing outliers, and the results showed that glaucoma was linked to a decreased risk of MI (OR: 0. 94, 95% CI: 0.89–0.99; P = 0.012). The effect estimates of glaucoma on MI by different MR methods are displayed in Figure 3A. The funnel plots is displayed in Figure 3B. The leave-one-out method revealed that no SNPs strongly affected the potential causal association between glaucoma and MI risk (Figure 3C).

**TABLE 2:** Heterogeneity and pleiotropy test for the associations of glaucoma with CVD.

Outcome	Heterogeneity test						Pleiotropy test		
	MR-Egger			Inverse variance weighted			MR-Egger		
	Q	Q_df	Q_pval	Q	Q_df	Q_pval	Egger intercept	se	pval
UAP	12.731	9	0.175	13.073	10	0.220	−0.012	0.025	0.634
CAD	9.306	8	0.317	10.245	9	0.331	0.015	0.017	0.395
HBP	31.092	8	1.35E-04	34.297	9	7.92E-05	0.003	0.003	0.390
MI	18.341	9	0.031	18.900	10	0.042	0.011	0.021	0.613
HF	14.607	8	0.067	15.730	9	0.073	0.013	0.017	0.455
IS	9.799	9	0.367	9.801	10	0.458	−0.001	0.016	0.971
PE	4.929	8	0.765	5.531	9	0.786	0.030	0.038	0.460
AF	14.090	9	0.119	20.431	10	0.025	0.027	0.013	0.075

UAP: unstable angina pectoris; CAD: coronary artery disease; HBP: high blood pressure; MI: myocardial infarct; HF: heart failure; IS: ischemic stroke; PE: pulmonary embolism; AF: atrial fibrillation; Q: Heterogeneity static Q; df: Degree of freedom; se: Standard error.

### Causal effects of CVD on glaucoma

**FIGURE 4:**
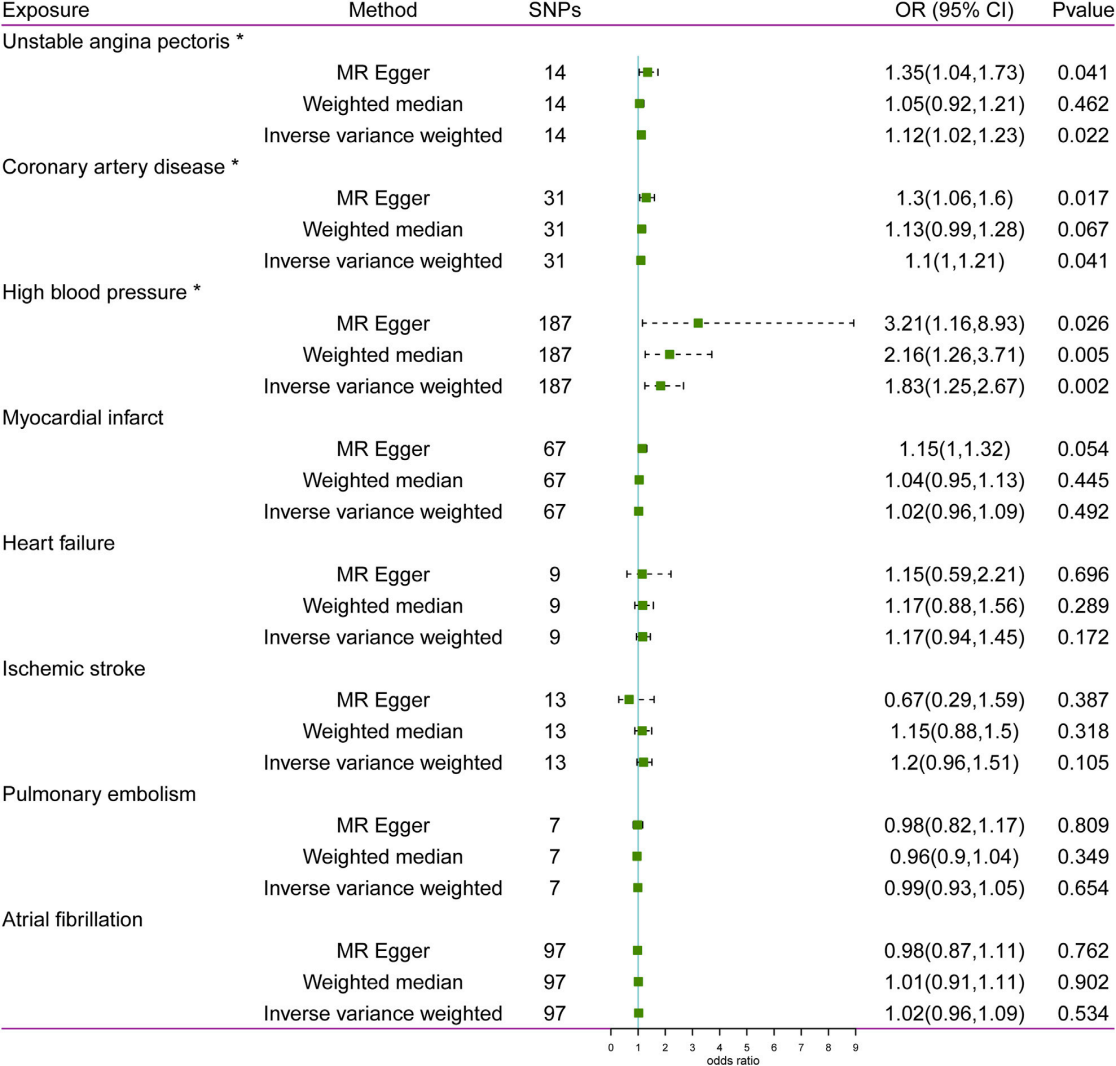
Associations between cardiovascular disease and risk of glaucoma using Mendelian randomization analysis. SNPs: Single-nucleotide polymorphisms, OR: Odds ratio, CI: Confidence interval.

**FIGURE 5:**
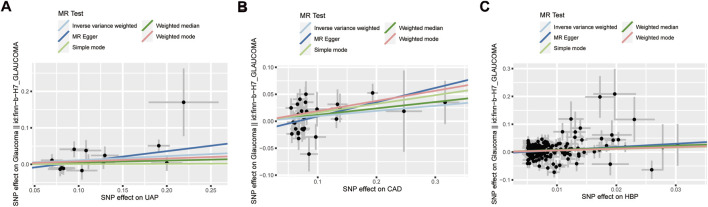
Scatter plot for the associations between UAP **(A)**, CAD **(B)**, as well as HBP **(C)** and glaucoma. UAP: Unstable angina pectoris; CAD: Coronary artery disease; HBP: High blood pressure; SNP: Single nucleotide polymorphism.

The statistical results of reverse-direction MR between CVD and glaucoma are depicted in Figure 4. We found that genetic susceptibility of UAP, CAD, and HBP was significantly linked to glaucoma (Figure 4). The Odds ratios (ORs) of UAP, CAD, and HBP were 1.12 (95% confidence interval (CI): 1.02–1.23; P = 0.022), 1.1 (95% CI: 1–1.21; P = 0.041), and 1.83 (95% CI: 1.25–2.67; P = 0.002), respectively, in the IVW model. However, no evidence was supported for the causal association between MI (OR: 1.02, 95% CI: 0.96–1.09; P = 0.492), HF (OR: 1.17, 95% CI: 0.94–1.45; P = 0.172), IS (OR: 1.2, 95% CI: 0.96–1.51; P = 0.105), PE (OR: 0.99, 95% CI: 0.93–1.05; P = 0.654), AF (OR: 1.02, 95% CI: 0.96–1.09; P = 0.534) and glaucoma. Noteworthily, the result of MR-Egger in HBP was consistent with the IVW method (Figure 5).

**FIGURE 6:**
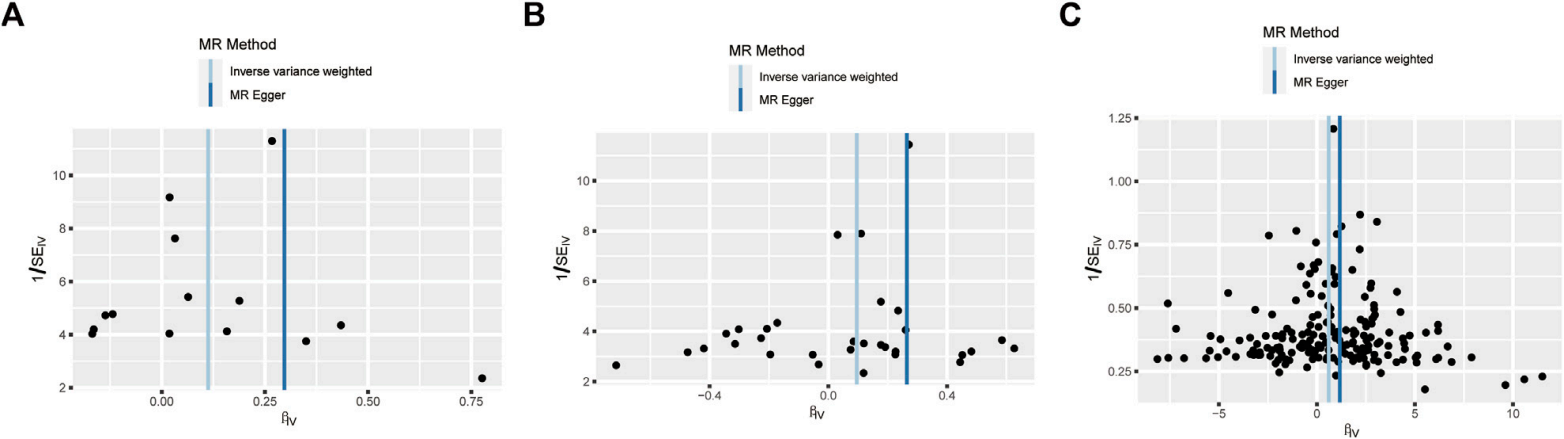
Funnel plot to assess the robustness. Unstable angina pectoris **(A)**, Coronary artery disease **(B)**, and High blood pressure **(C)**. MR: mendelian randomization; SE: standard error; IV: instrument variable.

MR-Egger regression and IVW analysis for UAP, CAD, MI, HF, IS, and PE revealed no heterogeneity among IVs. However, both MR-Egger regression (P = 0.005) and IVW analysis (P = 0.005) of HBP indicated the presence of heterogeneity among IVs (Table 3). The IVs in AF also exhibited heterogeneity (P = 0.0001). The MR-Egger regression intercept test indicated no horizontal pleiotropy among the IVs for CVD. Then, random effects IVM analysis demonstrated a causal association between HBP and glaucoma (P = 0.0017). After eliminating outliers (rs2032915 and rs73046792) in the MR-PRESSO analysis, genetic susceptibility of HBP was also significantly associated with glaucoma (P = 0.00015). The funnel plots of UAP, CAD, and HBP are displayed in Figure 6. Additionally, random effects IVM analysis showed that glaucoma susceptibility was unrelated to AF (P = 0.086). Furthermore, leave-one-out analysis demonstrated that the causal relationships between UAP, CAD, and HBP and glaucoma were not driven by any single SNP (Supplementary Figure S1).

**TABLE 3:** Heterogeneity and pleiotropy test for the associations of CVD with glaucoma.

Exposure	Heterogeneity test						Pleiotropy test		
	MR-Egger			Inverse variance weighted			MR-Egger		
	Q	Q_df	Q_pval	Q	Q_df	Q_pval	Egger intercept	se	pval
UAP	12.569	12	0.401	15.020	13	0.306	−0.023	0.015	0.152
CAD	35.806	29	0.179	39.748	30	0.110	−0.018	0.010	0.084
HBP	238.149	185	0.005	239.889	186	0.005	−0.005	0.004	0.246
MI	79.118	65	0.112	83.277	66	0.074	−0.012	0.006	0.069
HF	2.686	7	0.912	2.689	8	0.952	0.001	0.022	0.958
IS	16.348	11	0.129	19.144	12	0.085	0.036	0.026	0.197
PE	2.736	5	0.741	2.747	6	0.840	0.003	0.026	0.921
AF	154.450	95	0.000	155.349	96	0.000	0.004	0.005	0.459

UAP: unstable angina pectoris; CAD: coronary artery disease; HBP: high blood pressure; MI: myocardial infarct; HF: heart failure; IS: ischemic stroke; PE: pulmonary embolism; AF: atrial fibrillation; Q: Heterogeneity static Q; df: Degree of freedom; se: Standard error.

### Multivariable MR (MVMR) analysis

Based on the observed causal association between UAP, CAD, and HBP and glaucoma, further MVMR was performed to evaluate the causal effect of UAP, CAD, and HBP on glaucoma after adjusting for the underlying risk factors. The selected common risk factors of smoking, obesity, body mass index (BMI), and physical activity were validated whether there was the presence of causality with glaucoma in using MR analysis, and results showed that obesity and BMI were causally associated with glaucoma (Supplementary Table S1). Subsequently, obesity and BMI were included in the MVMR analysis, and the results showed that UAP and HBP were causally associated with glaucoma after controlling for the effects of obesity and BMI (Supplementary Table S2).

### SMR analysis

**FIGURE 7:**
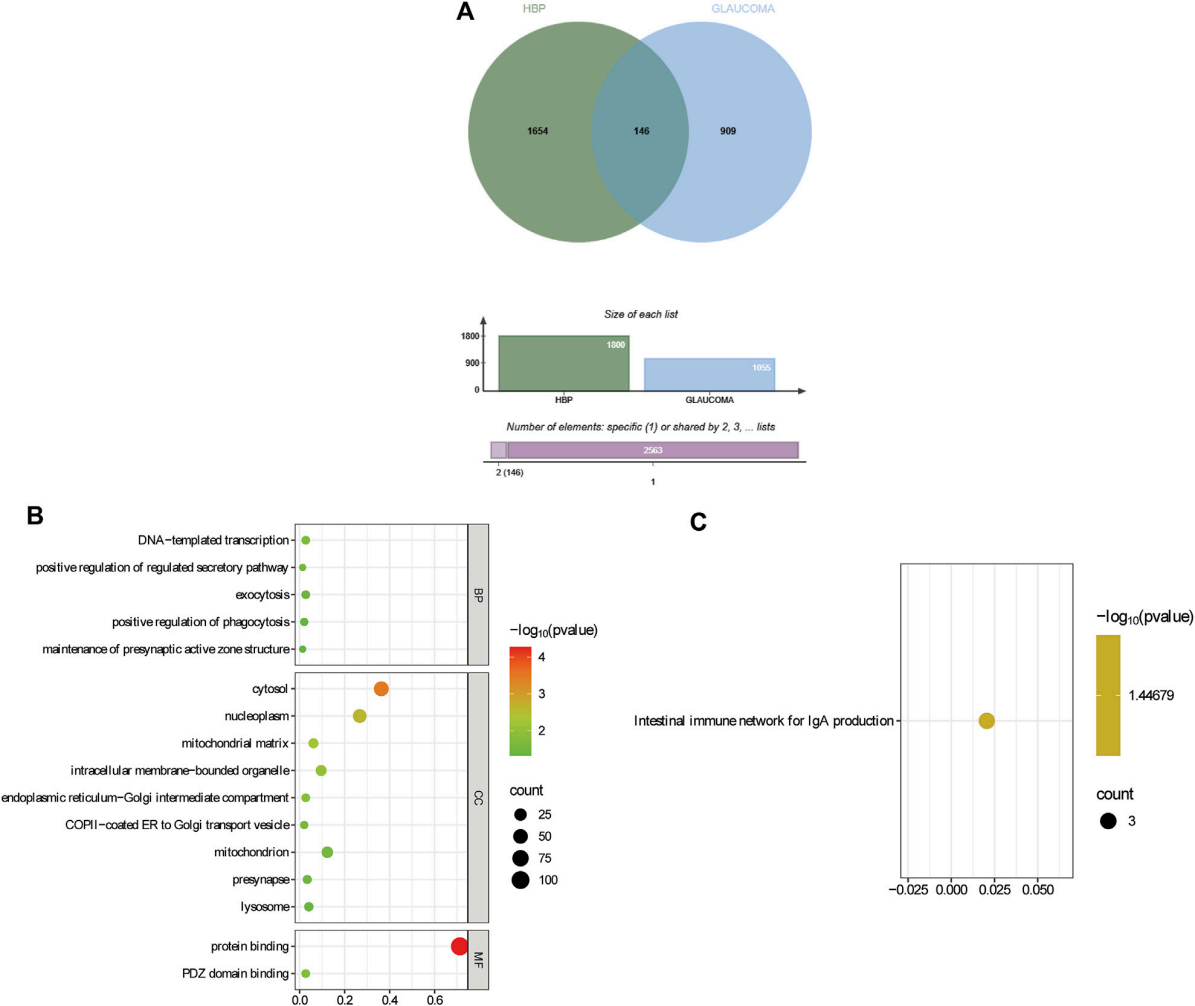
Identification and functional enrichment of common potential pathogenic genes for HBP and glaucoma. **(A)** Identification of common potential pathogenic genes. **(B)** GO enrichment of common potential pathogenic genes. **(C)** KEGG enrichment of common potential pathogenic genes.

**FIGURE 8:**
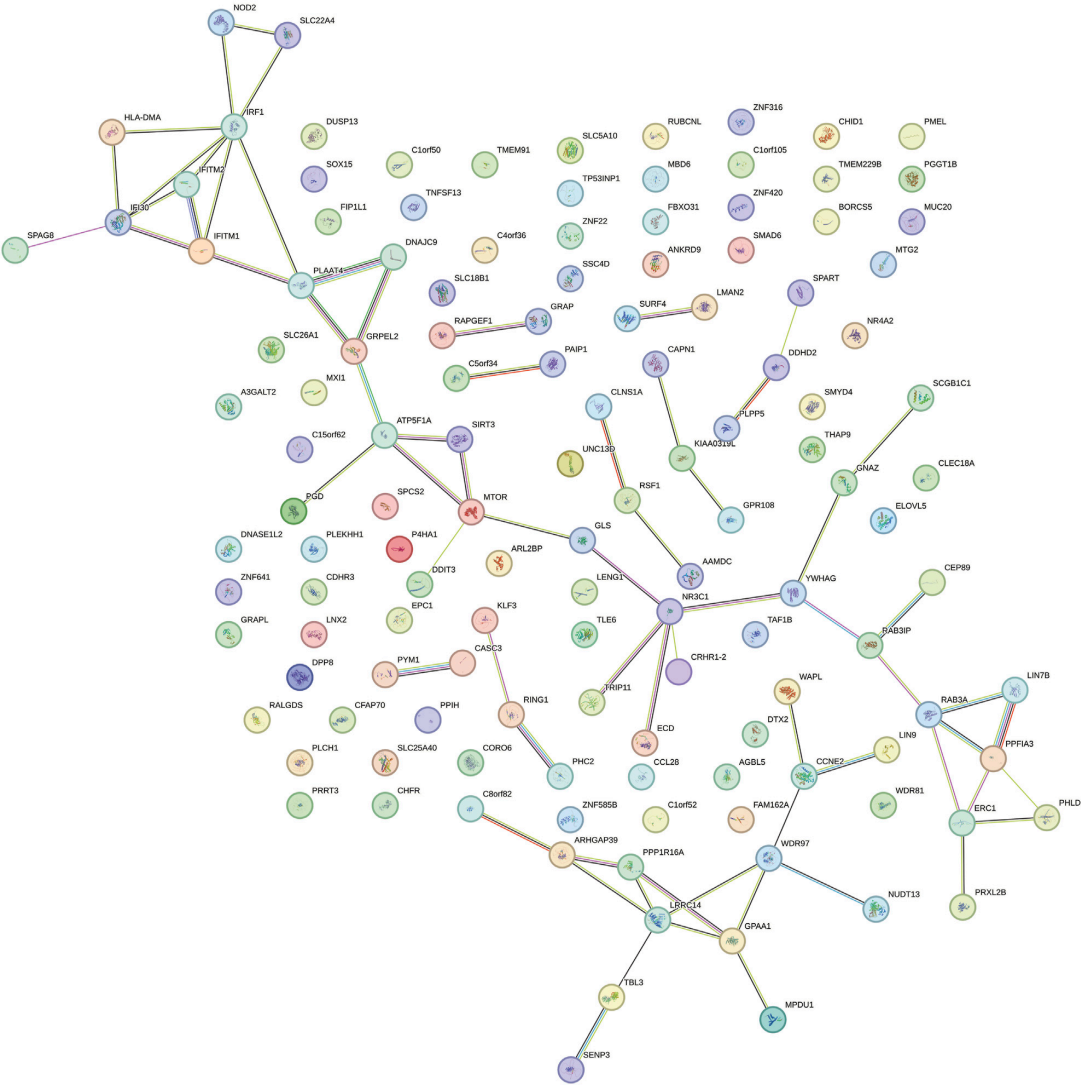
PPI network of proteins encoded by common potential pathogenic genes.

Through SMR analysis and filtering by P_SMR and P_HEIDI, 1800 and 1055 potential pathogenic genes (potential drug targets) related to HBP and glaucoma were identified, respectively. Among them, 146 genes were common potential pathogenic genes for HBP and glaucoma (Figure 7A). The GO enrichment analysis revealed that the common potential pathogenic genes were associated with 5 biological processes, 9 cellular component and 2 molecular functions (Figure 7B). Furthermore, only one pathway intestinal immune network for IgA production was enriched in KEGG (Figure 7C). The pathogenic genes enriched in the intestinal immune network for IgA production signaling pathway include HLA-DMA, TNFSF13 and CCL28. Subsequently, the PPI network was used to analyze the interactions among the proteins encoded by the common potential pathogenic genes (Figure 8). The number of interacting proteins for IRF1, IFI30, NR3C1 and LRRC14 was relatively abundant, suggesting that they may play a key role in the regulatory pathways of HBP and glaucoma.

## Discussion

In a longitudinal prospective study from UK Biobank, multivariable Cox regression analyses demonstrated that individuals with glaucoma were more susceptible to develop CVD than healthy controls [9]. In the population-based cohort study, cardiovascular mortality is elevated in individuals with glaucoma among 3,654 persons aged 49–97 years [33]. A meta-analysis indicated that MI, chronic ischemic heart disease, angina, and HBP were related to pseudoexfoliation, which is the common cause of OAG [34]. In an epidemiological study of patients with diabetes, glaucoma was positively correlated with MI and arterial hypertension [35]. Our study showed that glaucoma was associated with a lower risk of MI after removing outliers, while no evidence supported the causal effects between glaucoma and other CVDs. Previous MR studies have shown that the results are different before and after removing outliers [36–38]. Eliminating abnormal instrumental variables can optimize causal inference [39].

The precise mechanism through which glaucoma reduces the risk of MI remains unclear. Impaired ocular blood flow regulation is one of the factors affecting glaucomatous optic neuropathy [40, 41]. The self-regulating system of arteries, arterioles, and capillaries is essential for maintaining steady blood flow within the eye [40]. Nitric oxide (NO) and renin-angiotensin system (RAS) are one way to regulate the stabilization of intraocular blood flow [40, 42]. Polak et al. found that NO was increased in endothelial cells of glaucoma [43]. In the cardiovascular system, NO, acting on vascular smooth muscle, serves as a significant vasodilator and protector against MI-reperfusion injury [44]. RAS is an important fluid regulatory system in the body, playing a crucial role in regulating vascular tone and blood pressure [45]. High concentrations of angiotensin II (ANG-II), a major active peptide of the RAS, were observed in the aqueous humor of patients with POAG [46]. However, the conversion of ANG-(1–7) from ANG-II has been reported to have protective effects on the cardiovascular system [47]. Additionally, timolol, a nonselective beta-blocker, can not only alleviate OAG but also be used to prevent MI [48]. Therefore, we speculate that NO and RAS may be a bridge between glaucoma and MI.

Reverse MR analysis showed that UAP, CAD, and HBP were associated with an increased risk of glaucoma in our study. A prospective longitudinal study supported by Marshall et al. reported that CVD significantly increases the risk of glaucoma progression [8]. Moreover, having cardiovascular risk factors/disease is associated with higher risk of developing POAG [13]. The relationships among UAP, CAD, and glaucoma were not reported, while many studies were consistent with our findings in HBP. In a retrospective cohort study, Baek et al. reported that higher morning blood pressure surge was a significant independent predictor of visual field progression in normal-tension glaucoma patients with hypertension [49]. Another retrospective study demonstrated that HBP (HR: 1.056), stage 1 hypertension (HR: 1.101), and stage 2 hypertension (HR: 1.114) were associated with an elevated risk of glaucoma [50]. A regression discontinuity study found that HBP positively correlates with glaucoma, and antihypertensive therapy can significantly reduce the risk of glaucoma progression in a nationwide survey covering >2.6 million individuals [51]. Mechanically, increased IOP was the leading risk factor for glaucoma, and IOP is controlled by aqueous humor homeostasis [1, 40]. HBP could elevate IOP through increased secretion of aqueous humor and decreased outflow of aqueous humor to cause glaucoma [52]. While reverse MR suggested an association, our MVMR (adjusting for obesity/BMI) did not detect a robust causal effect of CAD on glaucoma. This indicates that the initial association observed in MR was likely mediated by shared metabolic risk factors. Furthermore, this discrepancy may also stem from residual confounding—CAD often clusters with complex cardiovascular comorbidities (dyslipidemia, endothelial dysfunction) not fully captured by our selected covariates (obesity, BMI). Notably, the lack of prior literature on the causal relationship between CAD and glaucoma, which further highlights the necessity of conducting in-depth research.

Through SMR analysis, 146 common potential pathogenic genes for HBP and glaucoma were identified. The enrichment of these genes in the intestinal immune network for IgA production pathway (involving HLA-DMA, TNFSF13, and CCL28) is particularly intriguing, as it hints at a potential crosstalk between gut immunity and ocular/vasculature homeostasis. Additionally, PPI network analysis showed that IRF1, IFI30, NR3C1 and LRRC14 have relatively abundant interacting proteins, suggesting that these genes might act as key regulatory nodes in the molecular pathways underlying HBP and glaucoma. Collectively, these findings may provide new insights into the shared pathogenic mechanisms of HBP and glaucoma, and the identified common genes and pathways might serve as potential therapeutic targets for further investigation.

The present study has several advantages. First, for the first time, the causal effects between glaucoma and CVD, including MI, HBP, CAD, UAP, HF, IS, PE, and AF, were investigated using bidirectional two-sample MR analysis. Secondly, IVW was used to assess the causal association, and weighted median and MR-Egger methods were utilized as the supplementary analysis. Thirdly, the evaluations of IV strength and sensitivity analysis were carried out to ensure the accuracy and reliability of the results. Some limitations should be noticed in this study. The subtypes of glaucoma and different stages of CVD were not classified, and the relationships need to be further explored. Then, this study included only European populations and may not be applied to other populations. In addition, stratified analysis, such as the subgroups based on sex, age, could not be conducted with aggregated information from the GWAS. In the future, we will collect a large number of clinical samples to verify the results of this study and conduct further stratified analyses.

## Conclusion

Conclusively, we found a causal effect between glaucoma and MI. In addition, reverse MR and MVMR suggested that UAP and HBP were the risk factors for glaucoma. However, the potential mechanisms among glaucoma, MI, UAP, and HBP require further investigation.

## Data Availability

Publicly available datasets were analyzed in this study. This data can be found here: The datasets presented in this study can be found in the IEU OpenGWAS project^1^. The accession numbers are finn-b-H7_GLAUCOMA, ebi-a-GCST90018932, ebi-a-GCST003116, ukb-b-14177, ebi-a-GCST90018877, ebi-a-GCST009541, ebi-a-GCST90018864, ebi-a-GCST006414 and ebi-a-GCST90013887, respectively.

## Footnotes

1
https://gwas.mrcieu.ac.uk

2
https://gwas.mrcieu.ac.uk/datasets/finn-b-H7_GLAUCOMA/

3
https://gwas.mrcieu.ac.uk/datasets/ebi-a-GCST90018932/

4
https://gwas.mrcieu.ac.uk/datasets/ebi-a-GCST003116/

5
https://gwas.mrcieu.ac.uk/datasets/ukb-b-14177/

6
https://gwas.mrcieu.ac.uk/datasets/ebi-a-GCST90018877/

7
https://gwas.mrcieu.ac.uk/datasets/ebi-a-GCST009541/

8
https://gwas.mrcieu.ac.uk/datasets/ebi-a-GCST90018864/

9
https://gwas.mrcieu.ac.uk/datasets/ebi-a-GCST006414/

10
https://gwas.mrcieu.ac.uk/datasets/ebi-a-GCST90013887/

## References

[1] Jayaram H Kolko M Friedman DS Gazzard G. Glaucoma: now and beyond. The Lancet (2023) 402:1788–801. 10.1016/s0140-6736(23)01289-8 37742700

[2] Steinmetz JD Bourne RRA Briant PS Flaxman SR Taylor HRB Jonas JB Causes of blindness and vision impairment in 2020 and trends over 30 years, and prevalence of avoidable blindness in relation to VISION 2020: the right to sight: an analysis for the global burden of disease study. The Lancet Glob Health (2021) 9:e144–e160. 10.1016/s2214-109x(20)30489-7 33275949 PMC7820391

[3] Jerrome S Joseph S Niranjana B Arkaprava M Lakshmanan P Balagiri S Agreement and reliability of transpalpebral tonometers with goldmann applanation tonometer: a systematic review and meta-analysis. Ophthalmol Glaucoma (2025) 8:242–56. 10.1016/j.ogla.2024.11.001 39542211

[4] Kang JM Tanna AP. Glaucoma. Med Clin North America (2021) 105:493–510. 10.1016/j.mcna.2021.01.004 33926643

[5] Hakim A Guido B Narsineni L Chen D-W Foldvari M. Gene therapy strategies for glaucoma from IOP reduction to retinal neuroprotection: progress towards non-viral systems. Adv Drug Deliv Rev (2023) 196:114781. 10.1016/j.addr.2023.114781 36940751

[6] Zhou X Xu J Zhang X Zhao Y Duan X. Causal relationships between gut microbiota and primary open-angle glaucoma: a Mendelian randomization and mediation analysis of glaucoma endophenotypes. Exp Eye Res (2024) 240:109788. 10.1016/j.exer.2024.109788 38218362

[7] Yuan R Liu K Cai Y He F Xiao X Zou J. Body shape and risk of glaucoma: a Mendelian randomization. Front Med (2022) 9:999974. 10.3389/fmed.2022.999974 36213644 PMC9538570

[8] Marshall H Mullany S Qassim A Siggs O Hassall M Ridge B Cardiovascular disease predicts structural and functional progression in early glaucoma. Ophthalmology (2021) 128:58–69. 10.1016/j.ophtha.2020.06.067 32730956

[9] Choi JA Lee SN Jung SH Won HH Yun JS. Association of glaucoma and lifestyle with incident cardiovascular disease: a longitudinal prospective study from UK biobank. Sci Rep (2023) 13:2712. 10.1038/s41598-023-29613-w 36792671 PMC9931750

[10] Deng Y Li Q Zhou F Li G Liu J Lv J Telomere length and the risk of cardiovascular diseases: a Mendelian randomization study. Front Cardiovasc Med (2022) 9:1012615. 10.3389/fcvm.2022.1012615 36352846 PMC9637552

[11] Lüscher TF. Cardiovascular diseases outside the heart: novel recommendations for pulmonary embolism and peripheral arterial disease. Eur Heart J (2020) 41:487–9. 10.1093/eurheartj/ehaa019 31960935

[12] Zhang J Tong H Jiang L Zhang Y Hu J. Trends and disparities in China's cardiovascular disease burden from 1990 to 2019. Nutr Metab Cardiovasc Dis (2023) 33:2344–54. 10.1016/j.numecd.2023.07.039 37596135

[13] Jung KI Kim YC Shin HJ Park CK. Nationwide cohort study of primary open angle glaucoma risk and cardiovascular factors among in Korean glaucoma suspects. Sci Rep (2025) 15:1952. 10.1038/s41598-025-85505-1 39809920 PMC11732984

[14] Cho HK Han JC Choi JA Chae JE Kim RB. Association between atrial fibrillation and the risk of glaucoma development: a 12-year nationwide cohort study. Eye (Lond) (2023) 37:2033–41. 10.1038/s41433-022-02274-1 36371604 PMC10333229

[15] Bennion JR Wise ME Carver JA Sorvillo F. Analysis of glaucoma-related mortality in the United States using death certificate data. J Glaucoma (2008) 17:474–9. 10.1097/IJG.0b013e318163bdbd 18794683

[16] Lin HC Chien CW Hu CC Ho JD. Comparison of comorbid conditions between open-angle glaucoma patients and a control cohort: a case-control study. Ophthalmology (2010) 117:2088–95. 10.1016/j.ophtha.2010.03.003 20570357

[17] Chen B Yan Y Wang H Xu J. Association between genetically determined telomere length and health-related outcomes: a systematic review and meta-analysis of Mendelian randomization studies. Aging Cell (2023) 22:e13874. 10.1111/acel.13874 37232505 PMC10352568

[18] Carter AR Sanderson E Hammerton G Richmond RC Davey Smith G Heron J Mendelian randomisation for mediation analysis: current methods and challenges for implementation. Eur J Epidemiol (2021) 36:465–78. 10.1007/s10654-021-00757-1 33961203 PMC8159796

[19] Meng Y Tan Z Su Y Li L Chen C. Causal association between common rheumatic diseases and glaucoma: a Mendelian randomization study. Front Immunol (2023) 14:1227138. 10.3389/fimmu.2023.1227138 37799717 PMC10550209

[20] Zhang J Chen X Zhu Y Wan S Hu S Yang Y. Investigating the causal relationship between sleep behaviors and primary open-angle glaucoma: a bidirectional two-sample Mendelian randomization study. Nat Sci Sleep (2024) 16:143–53. 10.2147/nss.S439274 38374869 PMC10876006

[21] Sakaue S Kanai M Tanigawa Y Karjalainen J Kurki M Koshiba S A cross-population atlas of genetic associations for 220 human phenotypes. Nat Genet (2021) 53:1415–24. 10.1038/s41588-021-00931-x 34594039 PMC12208603

[22] The CARDIoGRAMplusC4D Consortium, Goel A Won HH Hall LM Willenborg C Kanoni S A comprehensive 1,000 Genomes-based genome-wide association meta-analysis of coronary artery disease. Nat Genet (2015) 47:1121–30. 10.1038/ng.3396 26343387 PMC4589895

[23] Shah S Henry A Roselli C Lin H Sveinbjörnsson G Fatemifar G Genome-wide association and Mendelian randomisation analysis provide insights into the pathogenesis of heart failure. Nat Commun (2020) 11:163. 10.1038/s41467-019-13690-5 31919418 PMC6952380

[24] Nielsen JB Thorolfsdottir RB Fritsche LG Zhou W Skov MW Graham SE Biobank-driven genomic discovery yields new insight into atrial fibrillation biology. Nat Genet (2018) 50:1234–9. 10.1038/s41588-018-0171-3 30061737 PMC6530775

[25] Mbatchou J Barnard L Backman J Marcketta A Kosmicki JA Ziyatdinov A Computationally efficient whole-genome regression for quantitative and binary traits. Nat Genet (2021) 53:1097–103. 10.1038/s41588-021-00870-7 34017140

[26] Gan T Hu J Liu W Li C Xu Q Wang Y Causal association between anemia and cardiovascular disease: a 2-Sample bidirectional mendelian randomization study. J Am Heart Assoc (2023) 12:e029689. 10.1161/jaha.123.029689 37301769 PMC10356041

[27] Gao N Kong M Li X Zhu X Wei D Ni M The association between psoriasis and risk of cardiovascular disease: a mendelian randomization analysis. Front Immunol (2022) 13:918224. 10.3389/fimmu.2022.918224 35844511 PMC9278135

[28] Yang F Hu T He K Ying J Cui H. Multiple sclerosis and the risk of cardiovascular diseases: a mendelian randomization study. Front Immunol (2022) 13:861885. 10.3389/fimmu.2022.861885 35371017 PMC8964627

[29] Gao N Yu Z Fan Y Jiang X Hu T. Impact of negative emotions on upper gastrointestinal diseases: a mendel randomization study. (2024) 19:e0304121. 10.1371/journal.pone.0304121 38995968 PMC11244763

[30] Wang Z Lai W Zhong S. Investigating the causal relationship between human blood metabolites and coronary artery disease using two-sample Mendelian randomization. Nan Fang Yi Ke Da Xue Xue Bao (2021) 41:272–8. 10.12122/j.issn.1673-4254.2021.02.16 33624602 PMC7905253

[31] Xiao X Wu X Yi L You F Li X Xiao C. Causal linkage between type 2 diabetes mellitus and inflammatory bowel disease: an integrated Mendelian randomization study and bioinformatics analysis. Front Endocrinol (Lausanne) (2024) 15:1275699. 10.3389/fendo.2024.1275699 38313367 PMC10836595

[32] Song D-J Fan B Li G-Y. Blood cell traits and risk of glaucoma: a two-sample Mendelian randomization study. Front Genet (2023) 14:1142773. 10.3389/fgene.2023.1142773 37124610 PMC10130872

[33] Meyer NI. Open-angle glaucoma and cardiovascular mortality. Ophthalmology (2007) 114:392. 10.1016/j.ophtha.2006.09.007 17270687

[34] Siordia JA Franco J Golden TR Dar B. Ocular pseudoexfoliation syndrome linkage to cardiovascular disease. Curr Cardiol Rep (2016) 18:61. 10.1007/s11886-016-0738-5 27216841

[35] Nielsen NV. The prevalence of glaucoma and ocular hypertension in type 1 and 2 diabetes mellitus. An epidemiological study of diabetes mellitus on the island of falster, Denmark. Acta Ophthalmologica (1983) 61:662–72. 10.1111/j.1755-3768.1983.tb04357.x 6637428

[36] Wei H Ge H Qian Y Li B. Genetic determinants of inflammatory cytokines and their causal relationship with inflammatory disorders of breast: a two-sample Mendelian randomization study. Sci Rep (2025) 15:7300. 10.1038/s41598-025-91723-4 40025158 PMC11873064

[37] Jee Y Ryu M Sull JW. Alcohol consumption and cancer risk: two sample Mendelian randomization. Epidemiologia (2024) 5:618–26. 10.3390/epidemiologia5030043 39311360 PMC11417818

[38] Lee SH Lee JY Kim G Jung KJ Lee S Kim HC Two-sample mendelian randomization study of lipid levels and ischemic heart disease. Korean Circ J (2020) 50:940–8. 10.4070/kcj.2020.0131 32812402 PMC7515757

[39] Verbanck M Chen CY Neale B Do R. Detection of widespread horizontal pleiotropy in causal relationships inferred from Mendelian randomization between complex traits and diseases. Nat Genet (2018) 50:693–8. 10.1038/s41588-018-0099-7 29686387 PMC6083837

[40] Ekici E Moghimi S. Advances in understanding glaucoma pathogenesis: a multifaceted molecular approach for clinician scientists. Mol Aspects Med (2023) 94:101223. 10.1016/j.mam.2023.101223 39492376

[41] Wu X Konieczka K Liu X Chen M Yao K Wang K Role of ocular blood flow in normal tension glaucoma. Adv Ophthalmol Pract Res (2022) 2:100036. 10.1016/j.aopr.2022.100036 37846223 PMC10577859

[42] Kelly RA Kuhn MS Reina-Torres E Balasubramanian R Perkumas KM Li G Endothelial cell-specific postnatal deletion of Nos3 preserves intraocular pressure homeostasis *via* macrophage recruitment and NOS2 upregulation. J Clin Invest (2025) 135:e183440. 10.1172/jci183440 39932792 PMC11957705

[43] Polak K Luksch A Berisha F Fuchsjaeger-Mayrl G Dallinger S Schmetterer L. Altered nitric oxide system in patients with open-angle glaucoma. Arch Ophthalmol (2007) 125:494–8. 10.1001/archopht.125.4.494 17420369

[44] Russo I Barale C Melchionda E Penna C Pagliaro P. Platelets and cardioprotection: the role of nitric oxide and carbon oxide. Int J Mol Sci (2023) 24:6107. 10.3390/ijms24076107 37047079 PMC10094148

[45] Balakumar P Orayj KM Khan NA Shanmugam K Jagadeesh G. Impact of the local renin-angiotensin system in perivascular adipose tissue on vascular health and disease. Cell Signal (2024) 124:111461. 10.1016/j.cellsig.2024.111461 39389180

[46] Li H Cui H Ren J Wang D Zhao R Zhu S Elevated Angiotensin-II levels contribute to the pathogenesis of open-angle glaucoma *via* inducing the expression of fibrosis-related genes in trabecular meshwork cells through a ROS/NOX4/SMAD3 axis. Cell Transplant (2023) 32:09636897231162526. 10.1177/09636897231162526 36999649 PMC10068978

[47] Santos RAS Oudit GY Verano-Braga T Canta G Steckelings UM Bader M. The renin-angiotensin system: going beyond the classical paradigms. Am J Physiology-Heart Circulatory Physiol (2019) 316:H958–H970. 10.1152/ajpheart.00723.2018 30707614 PMC7191626

[48] Barnes J Moshirfar M. Timolol. StatPearls [Internet]. Treasure Island (FL): StatPearls Publishing (2024). 31424760

[49] Baek MS Song WK Kim KE Lee A Lee JY Shin JW Morning blood pressure surge and glaucomatous visual field progression in normal-tension glaucoma patients with systemic hypertension. Am J Ophthalmol (2023) 254:161–76. 10.1016/j.ajo.2023.06.014 37352910

[50] Lee JS Kim YJ Kim S Bae HW Kim SS Lee SW Increased risks of open-angle glaucoma in untreated hypertension. Am J Ophthalmol (2023) 252:111–20. 10.1016/j.ajo.2023.03.032 37030496

[51] Horwitz A Klemp M Jeppesen J Tsai JC Torp-Pedersen C Kolko M. Antihypertensive medication postpones the onset of glaucoma: evidence from a nationwide study. Hypertension (2017) 69:202–10. 10.1161/hypertensionaha.116.08068 27920127

[52] Leeman M Kestelyn P. Glaucoma and blood pressure. Hypertension (2019) 73:944–50. 10.1161/hypertensionaha.118.11507 30852921

