# Supplementary material for: Glaucoma and cardiovascular disease: a bidirectional two-sample Mendelian randomization analysis
Source: Exp Biol Med (Maywood). 2025 Oct 15;250:10610. doi: 10.3389/ebm.2025.10610 (PMC12568447; doi:10.3389/ebm.2025.10610)
Supplement: Supplementary file 2 [file Supplementaryfile1.docx]

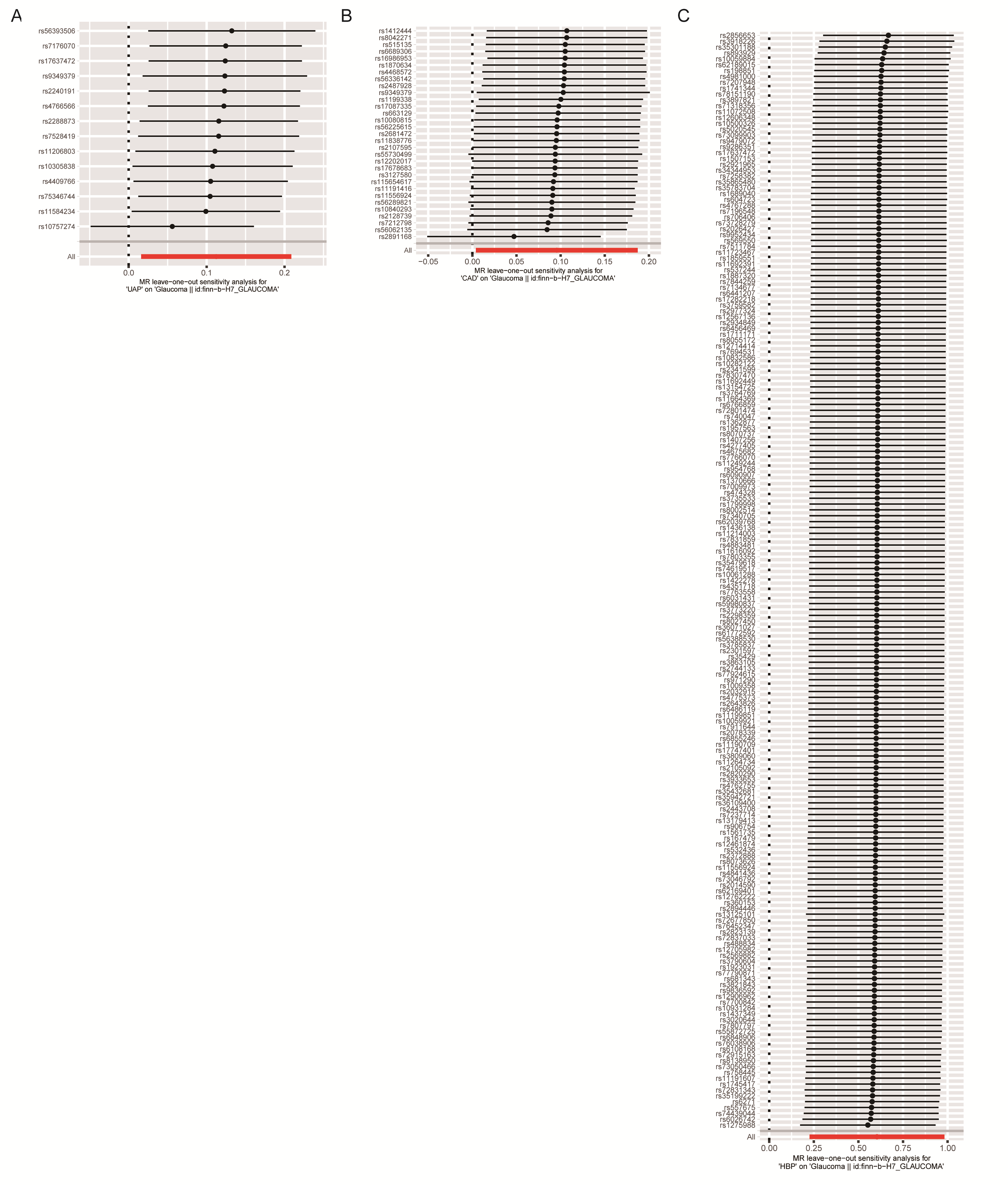


**Supplementary Figure 1**

Leave-one-out for the effect of UAP (A), CAD (B), as well as HBP (C), and glaucoma. UAP: Unstable angina pectoris; CAD: Coronary artery disease; HBP: High blood pressure; SNP: Single nucleotide polymorphism.
